# Association between social capital and mental health among older people living with HIV: the Sichuan Older HIV-Infected Cohort Study (SOHICS)

**DOI:** 10.1186/s12889-020-08705-6

**Published:** 2020-04-28

**Authors:** Jiayu Han, Peng Jia, Yuling Huang, Bo Gao, Bin Yu, Shifan Yang, Jun Yu, Jun Xiong, Chang Liu, Tian Xie, Peijie Dong, Chao Yang, Zixin Wang, Shujuan Yang

**Affiliations:** 1 Department of Health Related Social and Behavioral Science, West China School of Public Health and West China Fourth Hospital, Sichuan University, Chengdu, China; 2 Department of Land Surveying and Geo-Informatics, The Hong Kong Polytechnic University, Hong Kong, China; 3 International Initiative on Spatial Lifecourse Epidemiology (ISLE), Hong Kong, China; 4 Sichuan Center for Disease Control and Prevention, Chengdu, China; 5 Lu County Center for Disease Control and Prevention, Luzhou, China; 6 Pidu District Center for Disease Control and Prevention, Chengdu, China; 7 Department of epidemiology and statistics, School of Public Health, Southwest Medical University, Luzhou, China; 8 Centre for Health Behaviours Research, The Jockey Club School of Public Health and Primary Care, The Chinese University of Hong Kong, Hong Kong, China

**Keywords:** China, HIV, Mental health, Depression, Anxiety, Social capital

## Abstract

**Background:**

Mental health problems are common among older people living with HIV and associated with poorer health outcomes. Social capital is an important determinant of mental health problems but under-studied in this population. This study investigated the association between social capital and mental health problems among older people living with HIV in China.

**Methods:**

The study was based on the baseline data of a cohort study investigating mental health among older people living with HIV in Sichuan, China during November 2018 to February 2019. Participants were people living with HIV aged ≥50 years living in Sichuan province. Stratified multi-stage cluster sampling was used to recruit participants from 30 communities/towns; 529 out of 556 participants being approached completed the face-to-face interview. Social capital was measured by two validated health-related social capital scales: the Individual and Family scale and the Community and Society scale. Presence of probable depression (CES-D-10 score ≥ 10) and probable anxiety (GAD-7 score ≥ 5) were used as dependent variables. Two-level logistic regression models were applied to examine the association between social capital and probable depression/anxiety.

**Results:**

The prevalence of probable depression and probable anxiety was 25.9% (137/529) and 36.3% (192/529), respectively. After adjusting for significant covariates, the individual/family level of social capital was inversely associated with both probable depression (odds ratios (OR): 0.89, 95% CI: 0.84–0.93, *p* < 0.001) and probable anxiety (OR: 0.90, 95% CI: 0.86–0.95, *p* < 0.001). The community/society level social capital was associated with probable depression (OR: 0.91, 95% CI: 0.84–0.99, *p* < 0.001) but not probable anxiety (*p* > 0.05).

**Conclusions:**

Interventions building up social capital should be considered to improve mental health of older people living with HIV. Some useful strategies include cognitive processing therapy, improving community networking and engagement, and promoting social bonding with neighborhood.

## Background

Previous studies commonly define the age of older people living with HIV (PLWH) as 50 and above [1, 2]. Globally, the size of older PLWH has been rapidly increasing due to the advancement in the efficacy and coverage of antiretroviral therapy (ART) [3, 4]. For example, in the United States, the proportion of older PLWH over all PLWH was about 45% in 2014, and is projected to exceed 75% in 2030 [5]. In China, the number of newly diagnosed older PLWH has increased from 4751 in 2010 to 19,815 in 2017, accounting for 7.4 to 14.7% of all newly reported HIV cases in the corresponding years, respectively [6].

Living with HIV can be extremely challenging at any age, and older PLWH is a more vulnerable group than younger PLWH because aging and HIV infection can work interactively to reduce human immune response [7]. This makes older PLWH more susceptible to many chronic diseases including cardiovascular, lung, liver and renal diseases, cancers, and neuropsychiatric disorders. Older PLWH also have higher risk of diseases related to the acquired immune deficiency syndrome (AIDS), such as *Mycobacterium tuberculosis*, *Pneumocystis jirovecii* pneumonia, and toxoplasmosis [8, 9].

Mental health problems, such as depression and anxiety, are the most commonly reported comorbid conditions among PLWH [10]. Several studies have showed that prevalence of mental health problems among older PLWH ranged from 27.7 to 74.2% in different countries [8, 11–13]. For example, 27.7 and 39.9% of older PLWH in Brazil and the United States suffered from major depression, respectively [11, 12]. In China, the prevalence of depressive symptom was 74.2% among older PLWH [8], the rate is higher than that of their younger counterparts [8, 11–13], due to age-related reduction in immune responses, impaired physical function, reduced social support, or difficulties in coping with HIV-related stress [8, 14]. Therefore, more attention should be given to this older PLWH [15–17].

Some studies have examined the factors associated with mental health problems among PLWH, including disease-related characteristics (e.g., CD4 cell counts [18–20], time since diagnosis [19, 20], and duration on ART [19–22]]), and psychosocial factors (e.g., social capital, loneliness, ageism, and HIV-related stigma [16, 23]). Social capital, defined as the features of social organization such as trust, norms, and networks, is considered as fundamental driver of many other factors, which is especially true from a social epidemiology perspective [24–27]. Social capital can improve the efficacy of the society by facilitating the coordinated actions [27]. Some studies found an inverse association between social capital and mental health problems among PLWH [28–30]. However, such association may vary by age. For example, older PLWH, as a vulnerable population, are less likely to disclose their HIV sero-status to family, friends, relatives and health workers compared to their younger counterparts [8, 14]. This may make them feel more isolated and less supported by others, and thus lead to a different association between social capital and mental health problems. Nevertheless, to the best knowledge of the authors, none of the previous research has focused on the association between social capital and mental health problems among older PLWH. There is an urgent need to fill the knowledge gap in order to design effective and efficient social capital-related interventions to improve mental health and health equity among older PLWH.

Using data recently collected from two districts/counties with high HIV prevalence among the elderly in Sichuan Province of China [31–33], this study aimed to examine the association between social capital and mental health problems among older PLWH. The findings of this study will provide useful information for developing evidence-based interventions to build up social capital for preventing mental health problems and hence improving the quality of life among older PLWH [34]. It is hypothesized that stronger social capital at individual/family and/or community/society level would be associated with lower prevalence of probable depression and probable anxiety among older PLWH.

## Methods

### Study design

The data presented in this report was based on the baseline sample of an ongoing observational prospective cohort study investigating mental health problem among older PLWH in Sichuan province, China (the Sichuan Older HIV-infected Cohort Study, SOHICS). Questions asked by this study were parts of the cross-sectional baseline survey of the cohort, which was conducted from November 2018 to February 2019.

### Data collection

**Fig. 1:**
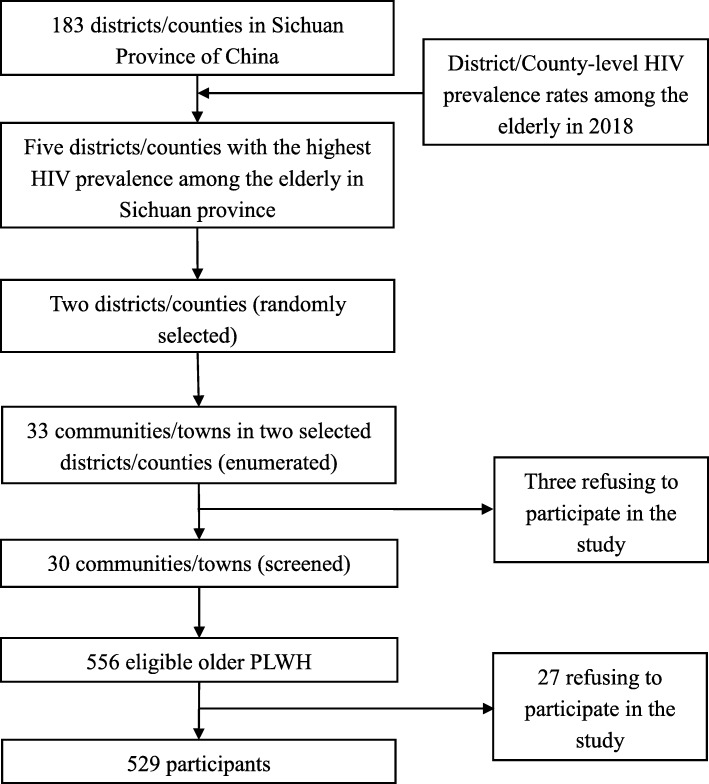
The sampling flowchart

As of 2018, there were 183 districts/counties in Sichuan. A stratified multi-stage cluster sampling design was used to select the study area of the SOHICS. First, two districts/counties were randomly selected from five districts/counties with the highest HIV prevalence among the elderly in Sichuan in 2018. Then, all sub-district (i.e., communities) or sub-county units (i.e., towns) within two selected districts/counties were contacted for participating in the study. According to the basic information system for AIDS Prevention and Treatment, all eligible subjects in those participating units were screened and invited to participate in the survey. The inclusion criteria for SOHICS participants were 1) receiving confirmatory HIV diagnosis, 2) at the age of 50 years or older at the time of diagnosis, 3) living in Sichuan for more than 5 years, and 4) receiving care and/or treatment in the local township health centers. Participants were excluded if they were found to have major psychiatric illness (e.g., schizophrenia and bipolar disorder) from their medical records, or were unable to communicate with the interviewers (Fig. 1).

According to the inclusion criteria and exclusion criteria, the participants were screened in the basic information system for AIDS Prevention and Treatment. Medical staff in township health centers phoned and briefed prospective participants about the study and confirmed their eligibility to participate in the study. Guarantees were made on anonymity and their right to quit at any time without being questioned, and that refusal would not affect their right to use any service in township health centers. Those who were interested in participating in the study were invited to pay a visit to their local township health centers. On site, trained interviewers briefed them again about the study and obtained their written informed consent. Ethics approval was obtained from the Ethics Committee West China School of Public Health and West China Fourth Hospital, Sichuan University.

A panel consisting of four epidemiologists, one health psychologist, and two staff in township health centers designed a questionnaire. It was tested and validated by conducting an anonymous face-to-face interview for 50 participants (25 randomly selected from each districts/counties) in private rooms in their local health centers. Their feedback was used to revise and finalize the questionnaire after panel discussion. Based on the pilot results, slight changes were made to improve readability of the questionnaire. No major change (e.g., removal of items) was made. Participants of the pilot study did not take part in the actual survey. This validated questionnaire was then used to aid with a 20-min anonymous face-to-face interview for all in private rooms in the same health centers. Participants were asked about their socio-demographic information, including age, sex, place of residence, ethnicity, education level, marital status, number of children, employment status, monthly personal income, duration of out migrating for work, and infection of spouse with HIV. Disease-related characteristics were extracted from their medical records, including route of HIV transmission, time since diagnosis, duration on ART, stage of HIV infection, and their most recent CD4 cell counts.

### Sample size planning of the SOHICS

Assuming the prevalence of probable depression/anxiety is 50% at the end of the follow-up period (12 months after baseline survey), the sample size of 400 older PLWH will confine the 95% confidence interval within +/− 4.9%. Given the assumption that prevalence of probable depression/anxiety in the reference group (e.g., those without a risk factor at baseline) to be 20–40% at Month 12, the sample size could detect the smallest odds ratios of 1.76 between those with and without such risk factor at baseline, with a statistical power of 0.8 and an alpha value of 0.05. Assuming the loss-to-follow-up rate to be 20% at Month 12, 500 older PLWH need to be recruited at baseline (PASS 11.0; NCSS; Kaysville; the United States).

### Measures

#### Social capital

The measurements of social capital used in this study were adapted from two scales in a validated Chinese version of Health-related Social Capital Measurement [35], i.e., the individual and family (IF) social capital scale and the community and society (CS) social capital scale. The IF scale had seven items. They were: 1) ‘You have many close contacts’, 2) ‘You have many social interaction with people other than your family members in the past month’, 3) ‘You always trust people who have social interaction with you’, 4) ‘You are satisfied with your marriage’, 5) ‘You always received emotional/financial/instrumental support from your spouse’, 6) ‘You always received emotional/financial/instrumental support from your relatives’, and 7) ‘You always received emotional/financial/instrumental from your close contacts in the last year’.

The CS scale also had seven items. They were: 1) ‘You frequently participated in activities organized by community organizations in the last year’, 2) ‘You always received support from community organizations in the last year’, 3) ‘You trust health organizations (i.e., hospitals and centers for diseases control and prevention) very much’, 4) ‘You trust community organizations very much’, 5) ‘You trust other governmental organizations very much’, 6) ‘You agree with the statement that hardworking people will be rewarded by the society’, and 7) ‘Do you agree with the statement that talented people will be recognized by the society’.

Response categories for both scales ranged from 1 (strongly disagree) to 5 (strongly agree), with a higher total score indicating the stronger social capital.

#### Mental health

We focused on two major types of mental health problems among older PLWH: depression and anxiety [10]. Depressive symptoms were measured using the Center for Epidemiologic Studies Depression (CES-D)-10 scale, which was constructed on the basis of self-reported responses to ten questions on whether, over the past week, participants had experienced symptoms associated with depression, such as worrying, sleeping difficulty and difficulty relaxing (Appendix 1). The CES-D-10 scale has a good test-retest reliability and predictive validity when compared with the original version of CES-D with 20 items [36], also with high sensitivity (97–100%) and specificity (84–93%) for screening major depression in middle-aged and older adults [37]. Responses were reported using a four-point Likert scale ranging from 0 (rarely or none of the time) to 3 (most or all of the time). The total score ranged from 0 to 30, with higher scores indicating more severe depressive symptoms. A cut-off score of 10 was used to define the presence of probable depression, which has been validated owning to minimizing false-positive results with little loss of sensitivity [36].

The Generalized Anxiety Disorder Scale (GAD-7) was used to screen participants for probable anxiety [38]. It recorded how often participants have suffered from seven problems over the past 2 weeks (Appendix 2). Responses were also reported using a four-point Likert scale ranging from 0 (not at all) to 3 (nearly every day). The total score ranged from 0 to 21, with higher scores indicating more severe anxiety symptoms. A cut-off score of 5 was suggested to define the presence of probable anxiety [39].

Both the CES-D-10 scale and the GAD-7 scale were validated in Chinese population [40, 41]. Both scales have been widely used in studies targeting PLWH and older adults in China [42–45].

#### Covariates

Covariates considered in this study included age, sex (male and female), place of residence (rural and urban), ethnicity (Han and minority), education level (illiterates, primary school, junior high school, and senior high school or above), marital status (unmarried, married and living with spouse, married but not living with spouse, and divorced and widowed), employment status (employed, retired, and unemployed), duration of out-migrating for work (none, ≤10 years and > 10 years), monthly personal income (none, < 1000 *yuan*, 1000–1999 *yuan*, and ≥ 2000 *yuan*) (one *yuan* was equivalent to about 0.14 US dollars at the time of conducting this study), having an HIV-infected spouse (yes, no, and do not have spouse), number of children (0, 1, 2, and ≥ 3), route of HIV transmission (sexual behaviour with spouse, sexual behaviour with a non-spouse opposite-sex partner, sexual behaviour with a same-sex partner, and blood transfusion), time since diagnosis (< 1 years, 1–3 years, and > 3 years), duration on ART (≤2 years and > 2 years), stage of HIV infection (HIV, AIDS, and missing), CD4 cell counts (< 200, 200–350, 351–500, > 500, and missing).

### Statistical analyses

Presence of probable depression (CES-D-10 score ≥ 10) and probable anxiety (GAD-7 score ≥ 5) were used as dependent variables. In line with many published studies, we dichotomized scores of the CES-D-10 and GAD-7 and used logistic regression models to investigate factors associated with probable depression and probable anxiety [46–48]. Two-level logistic regression models (level 1: towns/communities, level 2: individual older PLWH) were fit to analyze factors associated with the dependent variables. Random intercept models were used to allow the intercept of the regression model to vary across communities/towns, which could account for intra-correlated nested data. Two-level logistic regression models are commonly used in studies using similar sampling method as ours [49, 50]. Univariate logistic models were first used to examine the significance of the association between each covariate and outcome variable. Two sets of multivariate two-level logistic regression models were included in this study. The first set of multivariate logistic regression models controlled age and sex, as their relationships with mental health status are well established in previous studies [51]. Covariates with *p* < 0.10 in univariate analysis were adjusted in the second set of multivariate logistic regression models; similar approach was used in many published studies [52, 53].

Descriptive statistics, reliability analysis, and logistic regression embedded in the SPSS version 23.0 for Windows (SPSS, Inc., Chicago, IL, the United States) was used for data analysis, with *p* < 0.05 considered as statistically significant in final models. In addition, Cronbach’s alpha, ranging from 0 to 1, was used to assess the reliability of social capital scales. A Cronbach’s alpha of 0.6 or greater was considered acceptable [54, 55].

## Results

### Description of the participants

We conducted this study in two selected districts/counties, three of 33 communities/towns in these two units refused to participate in the survey. Therefore, 556 older PLWH from 30 communities/towns were contacted. Of them, 529 (95.1%) provided written informed consent and completed the interview, with 27 (4.9%) refusing to participate in the study. The mean scores of CES-D-10 and GAD-7 scales among 529 participants were 6.3 ± 6.1 and 4.0 ± 4.5, respectively. There were 25.9% (137/529) and 36.3% (192/529) of the participants showing the presence of probable depression and anxiety, respectively.

Table 1 showed study participants’ socio-demographic characteristics, indicators for HIV disease status, and social capital. The mean age of the participants was 63 years (SD = 7.2). Most of them were male (70.9%) and Han majority people (99.4%). Over half of the participants were married and living with spouse (51.1%), unemployed (60.3%), without a history of out-migrating for work (52.9%), having their permanent residency registered in the rural (77.1%), and with monthly income less than 2000 yuan (76.6%). The education level of the participants was low, as 16.2% were illiterates and 47.1% having attained primary schools. 40.4% had HIV-infected spouses, and 65.2% acquired HIV via sexual behaviours with an opposite sex non-spouse partner. The mean scores of the IF social capital scale and the CS social capital scale were 19.2 ± 4.9 and 23.9 ± 3.1, respectively (Table 2). Cronbach’s alphas of these two scales were 0.638 and 0.657, respectively, which were considered acceptable.

**Table 1:** Descriptive statistics of the participants (*n* = 529)

Variables	N	%
Socio-demographic characteristics		
Age group (years)		
50–59	166	31.4
60–69	262	49.5
≥ 70	101	19.1
Sex		
Male	375	70.9
Female	154	29.1
Ethnicity		
Han	526	99.4
Minority	3	0.6
Place of residence		
Rural	408	77.1
Urban	121	22.9
Education level		
Illiterate	86	16.2
Primary school	249	47.1
Junior high school	145	27.4
Senior high school or above	49	9.3
Marital status		
Unmarried	29	5.5
Married and living with spouse	270	51.1
Married but not living with spouse	78	14.7
Divorced/Widowed	152	28.7
Employment status		
Employed	137	25.9
Retired	73	13.8
Unemployed	319	60.3
Duration of out-migrating for work (years)		
None	280	52.9
≤ 10	105	19.8
> 10	144	27.2
Monthly personal income (RMB yuan)		
None	42	7.9
< 1000	228	43.2
1000-1999	135	25.5
≥ 2000	124	23.4
Having an HIV-infected spouse		
Yes	202	38.1
No	298	56.3
Do not have spouse	29	5.5
Number of children		
0	41	7.8
1	241	45.6
2	176	33.3
≥ 3	71	13.4
Disease-related characteristics		
Route of HIV transmission		
Sexual behavior with spouse	129	24.4
Sexual behavior with a non-spouse opposite-sex partner	345	65.2
Sexual behavior with a same-sex partner	28	5.3
Blood transfusion	27	5.1
Time since diagnosis (years)		
< 1	156	29.5
1–3	207	39.1
> 3	166	31.4
Duration on antiretroviral therapy (years)		
≤ 2	350	66.2
> 2	179	33.8
Stage of HIV infection		
HIV	249	47.1
AIDS	277	52.4
Missing	3	0.5
CD4 count in the most recent episode of testing (cls/μl)		
< 200	145	27.4
200–350	167	31.6
351–500	124	23.4
> 500	87	16.4
Missing	6	1.2

**Table 2:** Social capital of the participants (*n* = 529)

Variables	N	%
**Individual and family social capital** (% agree/strongly agree)		
You have many close contacts	170	32.1
You have many social interaction with people other than your family members in the past month	121	22.9
You always trust people who have social interaction with you	258	48.8
You are satisfied with your marriage	201	38.0
You always received emotional/financial/instrumental support from your spouse	221	41.8
You always received emotional/financial/instrumental support from your relatives	314	59.4
You always received emotional/financial/instrumental from your close contacts in the last year	75	14.2
**Community and society social capital** (% agree/strongly agree)		
You frequently participated in activities organized by community organizations in the last year	24	4.5
You always received support from community organizations in the last year	13	2.5
You trust health organizations (i.e., hospitals and centers for diseases control and prevention) very much	507	95.8
You trust community organizations very much	337	63.7
You trust other governmental organizations very much	493	93.2
You agree with the statement that hardworking people will be rewarded by the society	455	86.0
Do you agree with the statement that talented people will be recognized by the society	435	82.2

### Association between social capital and mental health

After adjusting for age and sex, the IF-based social capital was negatively associated with probable depression (OR: 0.88, 95% CI: 0.84–0.93, *p* < 0.001) and probable anxiety (OR: 0.91, 95% CI: 0.87–0.95, *p* < 0.001). The CS-based social capital was significantly associated with probable depression (OR: 0.91, 95% CI: 0.84–0.99, *p* < 0.05), but not with probable anxiety (OR: 0.96, 95% CI: 0.89–1.03, *p* > 0.05) (Table 4).

In univariate analyses, age, sex, education level, monthly personal income, route of HIV transmission, and time since HIV diagnosis were found to be potentially influential factors for both probable depression and anxiety (*p* < 0.1) (Table 3). Marital status, employment status and duration of ART were also potentially influential factors for probable anxiety but not for probable depression.

**Table 3:** Associations between covariates and the presence of mental health issues (measured as probable depression and anxiety) in binary regression models among participants (*n* = 529)

	OR (95% CI)	
	Probable depression	Probable anxiety
**Socio-demographics**		
Age group (years)		
50–59 (ref)	1	1
60–69	0.54 (0.34, 0.86)^*^	0.66 (0.43, 1.01)^*^
≥ 70	0.32 (0.17, 0.62)^*^	0.54 (0.31, 0.94)^*^
Sex		
Male (ref)	1	1
Female	2.81 (1.81, 4.35)^*^	2.46 (1.64, 3.71)^*^
Ethnicity		
Han (ref)	1	1
Minority	1.75 (0.14, 21.40)	1.23 (0.10,14.52)
Place of residence		
Rural (ref)	1	1
Urban	0.76 (0.45, 1.30)	0.87 (0.54, 1.39)
Education level		
Illiterate (ref)	1	1
Primary school	0.58 (0.32, 1.05)^*^	0.82 (0.48, 1.42)
Junior high school	0.67 (0.35, 1.27)	0.79 (0.43, 1.45)
Senior high school or above	0.30 (0.11, 0.80)^*^	0.26 (0.10, 0.65)^*^
Marital status		
Unmarried (ref)	1	1
Married and living with spouse	0.88 (0.32, 2.42)	1.67 (0.63, 4.48)
Married but not living with spouse	1.34 (0.45, 4.04)	2.30 (0.79, 6.68)
Divorced/widowed	1.48 (0.53, 4.12)	2.38 (0.88, 6.45)^*^
Employment status		
Employed (ref)	1	1
Retired	0.52 (0.23, 1.17)	0.50 (0.25, 1.01)^*^
Unemployed	1.41 (0.86, 2.31)	1.27 (0.81, 1.98)
Time of out-migrating for work (years)		
None (ref)	1	1
≤ 10 years	0.81 (0.47, 1.41)	0.77 (0.46, 1.27)
> 10 years	0.89 (0.53, 1.47)	0.86 (0.54, 1.36)
Monthly personal income (RMB)		
None (ref)	1	1
< 1000	0.42 (0.20, 0.87)^*^	0.63 (0.31, 1.29)
1000–1999	0.32 (0.14, 0.70)^*^	0.58 (0.27, 1.22)
≥ 2000	0.21 (0.09, 0.48)^*^	0.36 (0.17, 0.79)^*^
Having an HIV-infected spouse		
Yes (ref)	1	1
No	0.78 (0.51, 1.19)	0.90 (0.61, 1.3)
Do not have spouse	0.76 (0.28, 2.10)	0.47 (0.17, 1.27)
Number of children		
0 (ref)	1	1
1	1.08 (0.48, 2.43)	1.21 (0.57, 2.60)
2	0.82 (0.36, 1.89)	1.25 (0.58, 2.73)
3	0.62 (0.23, 1.65)	0.88 (0.36, 2.15)
**Disease-related characteristics**		
Route of HIV transmission		
Sexual behavior with spouse (ref)	1	1
Sexual behavior with a non-spouse opposite-sex partner	0.45 (0.28, 0.71)^*^	0.51 (0.33, 0.78)^*^
Sexual behavior with a same-sex partner	0.26 (0.08, 0.82)^*^	0.44 (0.17, 1.11)^*^
Blood transfusion	0.20 (0.05, 0.73)^*^	0.25 (0.08, 0.72)^*^
Time since diagnosis (years)		
< 1 (ref)	1	1
1–3	0.84 (0.51, 1.38)	0.90 (0.57, 1.43)
> 3	0.53 (0.30, 0.92)^*^	0.57 (0.34, 0.93)^*^
Duration of antiretroviral therapy (years)		
≤ 2 (ref)	1	1
> 2	0.76 (0.48, 1.19)	0.67 (0.45, 1.01)^*^
Stage of HIV infection		
HIV (ref)	1	1
AIDS	1.04 (0.69, 1.59)	0.88 (0.60, 1.28)
Missing	2.50 (0.20, 30.65)	1.28 (0.11, 15.25)
CD4 count in the most recent episode of testing (cls/μl)		
< 200	1	1
200–350	1.04 (0.60, 1.78)	1.01 (0.61, 1.65)
351–500	0.78 (0.43, 1.42)	0.95 (0.56, 1.63)
> 500	1.31 (0.70, 2.47)	1.42 (0.79, 2.54)
Missing	2.43 (0.40, 14.92)	2.92 (0.53, 16.08)

Boldfaced numbers indicate (*p < 0.1)

After adjusting for the abovementioned covariates with *p* < 0.1 in univariate analyses, the IF-based social capital was negatively associated with probable depression (OR: 0.89, 95% CI: 0.84–0.93, *p* < 0.001) and probable anxiety (OR: 0.90, 95% CI: 0.86–0.95, *p* < 0.001). The CS-based social capital was significantly associated with probable depression (OR: 0.91, 95%CI: 0.84–0.99, *p* < 0.05), but not with probable anxiety (OR: 0.96, 95% CI: 0.89–1.04, *p* > 0.05) (Table 4).

**Table 4:** Associations between social capital and the presence of mental health issues (measured as probable depression and anxiety) in two-level regression models among participants (*n* = 529)

	OR (95% CI)					
	Probable depression			Probable anxiety		
	Model 1	Model 2	Model 3	Model 4	Model 5	Model 6
Individual and family social capital scale	0.88 (0.84, 0.92)^***^	0.88 (0.84, 0.93)^***^	0.89 (0.84, 0.93)^***^	0.91 (0.87, 0.95)^***^	0.91 (0.87, 0.95)^***^	0.90 (0.86, 0.95)^***^
Community and society social capital scale	0.89 (0.83, 0.97)^**^	0.91 (0.84, 0.99)^*^	0.91 (0.84, 0.99)^*^	0.95 (0.88, 1.01)	0.96 (0.89, 1.03)	0.96 (0.89, 1.04)

Models 1 and 4: including only social capital measure as an independent variable

Models 2 and 5: adjusted for age and sex

Models 3: adjusted for age, sex, education level, monthly personal income, route of HIV transmission, and time since diagnosis

Model 6: adjusted for age, sex, education level, marital status, employment status, monthly personal income, route of HIV transmission, time since diagnosis and duration of antiretroviral therapy

Boldfaced numbers indicate statistical significance (^*^p < 0.05, ^**^p < 0.01, ^***^p < 0.001)

## Discussion

This study brought special attention to the importance of roles played by social capital in mental health of an understudied vulnerable population – older PLWH, who have more severe mental health problems than PLWH in other age groups. To our knowledge, this is the first study exploring the association between social capital and mental health problems among older PLWH, especially in China. We found that individual and family social capital was negatively associated with probable depression and anxiety, and community and society social capital was negatively associated with probable depression.

As identified by a systematic review, moderate evidence supported an inverse association between social capital and mental health problems among children. Studies targeting PLWH of younger age also showed a similar association between depressive symptoms and social capital [28–30]. Similar to these studies, our study showed that stronger social capital was associated with lower likelihood of mental health problems among older PLWH. In particular, family social network, and trust/intensity of relationship with family members and others played important roles in mental health wellbeing among older PLWH, as the individual and family social capital scale score representing better social capital were significantly associated with lower probable depression and probable anxiety. The traditional Chinese culture valued the support from family members. Family members usually form a very close social circle, and a strong attachment to each other. Previous studies among PLWH in China showed that patient’s family plays an important role in providing PLWH with mental support and companionship [56], which could help them reduce fear of ridicule, discrimination and death [57]. In addition, our study showed that more community participation/support and trust of the society were associated with lower likelihood of probable depression. This finding is consistent with those of previous studies conducted in Africa among PLWH [58, 59], which support that living in a trusting social environment that alleviates daily stressors and promotes good health behaviour may be protective against mental health problems. Therefore, interventions building social capital may be useful to prevent mental health problems in this group. Some commonly used strategies include cognitive processing therapy, improving community networking and engagement (e.g., increase involvement in cultural/religion activities, improvement in employment/training opportunities), and promoting social bonding with neighborhood.

There has not been any consensus about pathways and causality between social capital and mental health problems. Some studies suggested that presence of mental health problems (e.g., depression) would lead to social withdrawal, and related symptoms may cause inability to accurately perceive the availability of the resources, which would lead to weaker social capital [60]. Others argued that weaker social capital would hinder the access to resources, and may serve as a pathway to or aggravate pre-existing mental health problems [61]. For older PLWH, social capital can enhance the diffusion of AIDS-related health information and thus foster norms of healthy behaviours that could improve mental health [62]. Social capital is also thought to provide psychosocial support that can reduce HIV-related stress and thus improve mental health [62]. A recent published systematic review showed that social capital interventions were effective in improving subjective well-being, social support, resilience, sense of community and quality of life, and reducing loneliness and depression [63]. More programs should be designed in the future to test the feasibility and efficacy of social capital interventions among older PLWH.

To our knowledge, this was one of the first studies looking at association between different levels of social capital and mental health problems among older PLWH. The stratified multi-level cluster sampling design increased the representativeness of the study sample. However, this study also had several limitations. First, the cross-sectional baseline data prevented us from making strong causal inferences between social capital and mental health. The mechanisms underlying how social capital may influence mental health remain unknown and to be explored after follow-ups [64, 65]. Recall bias may exist, as data were self-reported. The modern approaches of collecting data, such as smartphone-based applications, could be used in future longitudinal studies to collect data in a more frequent, real-time manner, probably improving the reliability of the measurements of social capital scales [66, 67]. Second, more aspects of mental health, such as stress, should be measured in future in order to fully understand influences of social capital on mental health problems among older PLWH. Third, an employment-related social capital scale could also be used to measure social capital among those employed participants. Fourth, some influential factors established in previous literature, such as CD4 cell counts [18–20], may not be fully controlled in this study, partly due to the limited number of participants. The present study area would be expanded to include a new district/county with high prevalence of HIV in the first follow-up of the SOHICS, so a larger sample size was to be recruited to enable more well-established covariates to be controlled for in future efforts. Fifth, our study was conducted in areas with high HIV prevalence, so cautious should be taken when generalizing the findings to other areas or contexts. Moreover, the association found in our study area may vary across Sichuan and even China due to socio-cultural differences over geography. Follow-ups and other future efforts that would recruit participants from a wider region, with more variation among participants, would help with a more comprehensive understanding of the influences of social capital on mental health issues, such as the interactive effects of social capital and other determinants.

## Conclusions

This study revealed an inverse association between social capital and two mental health problems among older PLWH in Sichuan Province of China. It implied that interventions building up social capital should be considered to improve their mental health, such as cognitive processing therapy, improving community networking and engagement, and promoting social bonding with neighborhood. Future programs should test the feasibility and efficacy of social capital-related interventions in this vulnerable population. More importantly, this study drew much attention to this understudied issue in vulnerable populations, which have enabled us to obtain more support from multiple stakeholders including the central and local governments for expanding this effort to a larger sample in a larger region. Longitudinal data will be used to better understand the causal association between social capital and mental health in the population with the greatest need.

## Data Availability

The datasets used and analysed during the current study are available from the corresponding author on reasonable request.

## Appendix 1

### The Center for Epidemiologic Studies Depression (CES-D)-10 items

**Table:**

Item 1	I was bothered by things that usually don’t bother me.
Item 2	I had trouble keeping my mind on what I was doing.
Item 3	I felt depressed.
Item 4	I felt that everything I did was an effort.
Item 5	I felt hopeful about the future.
Item 6	I felt fearful.
Item 7	My sleep was restless.
Item 8	I was happy.
Item 9	I felt lonely.
Item 10	I could not “get going”.

## Appendix 2

### The Generalized Anxiety Disorder (GAD)-7 items

**Table:**

Item 1	Feeling nervous, anxious or on edge
Item 2	Not being able to stop or control worrying
Item 3	Worrying too much about different things
Item 4	Trouble relaxing
Item 5	Being so restless that it is hard to sit still
Item 6	Becoming easily annoyed or irritable
Item 7	Feeling afraid as if something awful might happen

## Abbreviations

PLWH: People living with HIV
IF: Individual and family
CS: Community and society
SOHICS: The Sichuan Older HIV-Infected Cohort Study
AIDS: Acquired immune deficiency syndrome
ART: Antiretroviral therapy
CES-D-10: The Center for Epidemiologic Studies Depression-10 scale
GAD-7: The Generalized Anxiety Disorder Scale
